# Metacognitive training for delusions (MCTd): effectiveness on data-gathering and belief flexibility in a Chinese sample

**DOI:** 10.3389/fpsyg.2015.00730

**Published:** 2015-06-15

**Authors:** Suzanne Ho-Wai So, Arthur P. Chan, Catherine Shiu-Yin Chong, Melissa Hiu-Mei Wong, William Tak-Lam Lo, Dicky Wai-Sau Chung, Sandra S. Chan

**Affiliations:** ^1^Department of Psychology, The Chinese University of Hong KongHong Kong, China; ^2^Early Intervention Service for First Episode Psychosis, Kwai Chung HospitalHong Kong, China; ^3^Tai Po HospitalHong Kong, China; ^4^Department of Psychiatry, The Chinese University of Hong KongHong Kong, China

**Keywords:** psychosis, delusions, metacognitive, reasoning, training, psychological intervention, flexibility, Chinese

## Abstract

Metacognitive training (MCT) was developed to promote awareness of reasoning biases among patients with schizophrenia. While MCT has been translated into 31 languages, most MCT studies were conducted in Europe, including newer evidence recommending an individualized approach of delivery. As reasoning biases covered in MCT are separable processes and are associated with different symptoms, testing the effect of selected MCT modules would help to develop a targeted and cost-effective intervention for specific symptoms and associated mechanisms. This study tested the efficacy of a four-session metacognitive training for delusions, MCTd (in Traditional Chinese with cultural adaptations, provided individually), as an adjunct to antipsychotics in reducing severity and conviction of delusions, jumping to conclusions (JTC) bias and belief inflexibility. Forty-four patients with delusions were randomized into the MCTd or the wait-list control condition. Patients on wait-list received the same MCTd after 4 weeks of treatment as usual (TAU). Assessment interviews took place before and after the treatment, and at 4-week follow-up. There was an additional baseline assessment for the controls. JTC and belief flexibility were measured by the beads tasks and the Maudsley Assessment of Delusions Scale. Attendance rate of the MCTd was satisfactory (84.5%). Compared to TAU, there was a greater reduction in psychotic symptoms, delusional severity and conviction following MCTd. There was a large treatment effect size in improvement in belief flexibility. Improvement in reaction to hypothetical contradiction predicted treatment effect in positive symptoms and delusions. JTC bias was reduced following MCTd, although the treatment effect was not significantly larger than TAU. Our results support the use of process-based interventions that target psychological mechanisms underlying specific psychotic symptoms as adjuncts to more conventional approaches.

## Introduction

Psychosis is a complex condition encompassing a range of symptoms (van Os et al., 1999; Bürgy, 2008; Demjaha et al., 2009). In view of heterogeneity of illness experience and treatment needs, psychosocial intervention programs for psychosis have adopted a modular approach (e.g., Addington and Gleeson, 2005; So et al., 2013), including more broad-based interventions for all patients with psychosis (e.g., psychoeducation and support groups, Ascher-Svanum and Krause, 1991; Lincoln et al., 2007; Castelein et al., 2008; Rummel-Kluge and Kissling, 2008; Froböse et al., 2014), and more focused interventions for specific psychotic symptoms (e.g., cognitive-behavioral therapy for voices and delusions, Morrison and Renton, 2001; Trower et al., 2004; Freeman et al., 2008; Hagen et al., 2011; Thomas et al., 2011). Large-scale randomized-controlled studies and meta-analyses have found cognitive-behavioral therapy for psychosis (CBTp) to be effective in reducing treatment-resistant psychotic symptoms as well as depression in association with psychosis (Tarrier and Wykes, 2004; Garety et al., 2008; Wykes et al., 2008; National Institute of Clinical Excellence [NICE], 2014; Turner et al., 2014). However, effect sizes (0.2–0.4) were modest, especially in better-controlled trials, and there is a call to improve CBTp by focusing more on the cognitive mechanisms of change (Velligan, 2009; Garety et al., 2014; Jauhar et al., 2014).

Research has shown that a number of reasoning processes contribute to the development and maintenance of delusions (see reviews by So et al., 2010; Garety and Freeman, 2013; Freeman and Garety, 2014). In contrast to neurocognitive deficits such as memory and attention, these processes pertain to the way individuals gather and process information towards making a decision or interpreting experiences. These processes include the ‘jumping to conclusions’ (JTC) data-gathering bias, lack of belief flexibility, externalizing attributional style, and theory of mind deficit. JTC is a tendency for individuals to make decisions based on insufficient data-gathering, which is usually measured using the Beads task (Garety et al., 1991). Newer JTC studies have shown that patients with psychosis are not only hastier in data-gathering than non-clinical individuals, but also more confident in their decisions (Moritz et al., 2006; Kircher et al., 2007), suggesting the possibility that over-confidence in errors maintains delusional beliefs (Moritz et al., 2013). Lack of belief flexibility is a difficulty in appreciating that one may be mistaken of his/her delusional belief, and in accommodating alternative explanations (Freeman et al., 2004).

According to Moritz et al. (2010), the reasoning biases that have been identified in psychosis are separate factors and should be targeted independently in intervention. Our systematic review (So et al., 2010) further confirmed that different reasoning processes are related to different symptoms of psychosis. While JTC and lack of belief flexibility are closely associated with delusions, theory of mind deficit relates more to disorganization and negative symptoms than to positive symptoms, and attributional style may be related to overall psychopathology rather than to specific symptoms. In addition, JTC and lack of belief flexibility are associated with the strength of delusions (i.e., conviction), and predict treatment response (So et al., 2010).

Although JTC and lack of belief flexibility did not improve in response to antipsychotics (Peters and Garety, 2006; So et al., 2012), research suggests that they are potential moderating and mediating variables which, when effectively ameliorated, may promote improvement in delusions (Garety et al., 1997, 2014; Menon et al., 2008; Dudley et al., 2013; Sanford et al., 2013; So et al., 2014). As suggested by Freeman (2011), evaluating the effect of process-based interventions on clearly defined etiological factors and subsequent change in delusions provides a rigorous methodology for advancing understanding of the causes of delusions.

Metacognitive training (MCT) aims at raising patients’ awareness of metacognitive disturbances so as to improve their repertoire of problem solving and to prevent relapse (Moritz and Woodward, 2007; Moritz et al., 2014a). Although MCT and CBTp both aim to improve psychotic symptoms and prevent relapse, their therapeutic components and processes are different. CBTp includes active therapy techniques as follows: enhancing self-regulatory strategies, development of a personal model of psychosis and relapse, work on reinterpreting the meaning of delusional beliefs and hallucinations, schema work, and relapse prevention (Dunn et al., 2012). Unlike CBTp, MCT does not emphasize patients’ idiosyncratic belief systems or their views about psychosis. Rather than focusing directly on the content of patients’ delusional beliefs and their associated emotions, MCT takes the ‘back door approach,’ identifying and discussing at length the underlying cognitive processes that contribute to the delusional interpretations of experiences (Moritz et al., 2011a; Kumar et al., 2014). This non-directive approach in addressing delusional beliefs and underlying reasoning biases is considered to be less threatening to participants and potentially helpful in minimizing treatment resistance (Moritz et al., 2014a).

The original MCT program consists of eight sessions. Each session focuses on one of the following cognitive biases: ‘JTC’ bias, attributional biases, bias against disconfirmatory evidence, social cognition (empathy and theory of mind), over-confidence in errors, and depressive cognition (Moritz and Woodward, 2007). Designed to be delivered as a psychoeducation group, MCT sessions are highly structured and manualized (http://www.uke.de/mct). Each MCT session consists of the following components:

(i) introduction and normalization of a specific reasoning bias, illustrated by historical events and daily life examples;
(ii) enhancing experiential learning about the bias by engaging group members in a series of exercises using cartoons, artwork or non-personalized daily-life events; and
(iii) linking the bias to problematic coping in general and symptoms of psychosis in particular.

An individualized format of MCT, the MCT+, has been subsequently developed as an extension to include generation of an illness model and a recovery plan, as well as intervention for negative symptoms (Vitzthum et al., 2014). Compared with MCT, MCT+ takes longer (i.e., 10 sessions) and includes treatment components more comparable to CBTp (Moritz et al., 2011b).

Since its advent, efficacy of the MCT has been put to test in 17 small-to-medium sized studies, including randomized controlled trials (see review by Moritz et al., 2014a; see also Gawęda et al., 2015). MCT had shown superior effects over various control conditions, including treatment as usual (TAU; in most trials) and active controls such as CogPack and supportive therapy, with effect sizes ranging from small to large on positive symptoms including delusions (Moritz et al., 2014a). In the MCT trial that consisted of patients with delusions only (Kumar et al., 2010), there was a medium-to-large treatment effect on PANSS positive score, but change in delusions or delusional dimensions was not reported. There is emerging evidence supporting a longer-term efficacy of MCT, with a reduction in positive symptoms sustained up to 3 years after intervention (Favrod et al., 2014; Moritz et al., 2014b).

The MCT has been translated into 31 languages, and there is new evidence of the efficacy of MCT beyond Germany, where it originates (Kumar et al., 2010; Gawęda et al., 2015). The Traditional Chinese version of the MCT has recently been tested for the first time (Lam et al., 2014). However, this trial focused on cognitive insight and self-efficacy only, without a report of symptom changes.

Treatment efficacy on the JTC bias had been reported in four group-based MCT trials (Aghotor et al., 2010; Moritz et al., 2011a, 2014b; Gawęda et al., 2015), with inconsistent findings. However, studies that tested individualized training or blended versions appeared to have more positive results for JTC. Moritz et al. (2011b) reported that the combined MCT/MCT+ intervention yielded a superior improvement in severity and conviction of delusions as well as JTC bias than an active control condition. Ferwerda et al. (2010) also reported a significant reduction in paranoid delusions, data-gathering and cognitive flexibility following MCT+. In Waller et al. (2011) and Garety et al. (2014), patients with delusions had significant improvements in JTC and delusional conviction following the one-session Maudsley Review Training Program (MRTP). The MRTP is a computerized treatment program with a particular focus on JTC and belief flexibility and their links with delusions. Unlike MCT, MRTP incorporates material intended to be salient and personally relevant, and encourages use of strategies through interactive tasks. The MRTP studies also provided the only evidence for change in belief flexibility following MCT-based intervention, although the change in belief flexibility was evident only after 2 weeks of post-treatment homework exercises whereas JTC change took place immediately after treatment. The success of the MRTP trial suggests that selected modules of the MCT can be delivered with efficacy that is clinically and statistically significant. It also supports a combination of MCT elements and individualized applications of the learnt skills.

The present study examined the efficacy of a brief four-session package of the Traditional Chinese MCT for delusions (MCTd) in reducing severity and conviction of delusions, JTC and belief inflexibility. As the study aimed to examine treatment outcome in delusions, only the modules related to data-gathering and belief flexibility were included in MCTd. Based on findings from Waller et al. (2011) and Garety et al. (2014), MCTd was delivered individually.

Key hypotheses of the study were as follows:

(1) The four-session package of MCTd will be considered feasible, acceptable and useful by patients with delusions
(2) There will be a greater reduction in severity and conviction of delusions after MCTd than wait-list
(3) There will be a greater improvement in data-gathering and belief flexibility after MCTd than wait-list
(4) Treatment effect on delusions will be mediated by improvement in cognitive biases (JTC and belief flexibility).

## Materials and Methods

### Clinical Ethics

Ethics approval and site approval were obtained from the Kowloon West Cluster Research Ethics Committee [Reference number: KW/EX-13-062(62-14)] and the Joint Chinese University of Hong Kong – New Territories East Cluster Clinical Research Ethics Committee [Reference number: CRE-2013.035].

### Participants

Participants were outpatients who presented with delusions [scoring 4 or above on at least one of the delusion items on the Positive and Negative Syndrome Scale (PANSS), Kay et al., 1987] and had a casenote diagnosis of a schizophrenia spectrum disorder. Participants were on antipsychotics for at least a month, and were recruited from psychiatric clinics of the Hong Kong Hospital Authority. Patients with drug-induced psychosis or organic psychosis, patients with intellectual disability, and patients with a primary diagnosis of substance misuse were excluded.

### Design

#### Procedure

**FIGURE 1:**
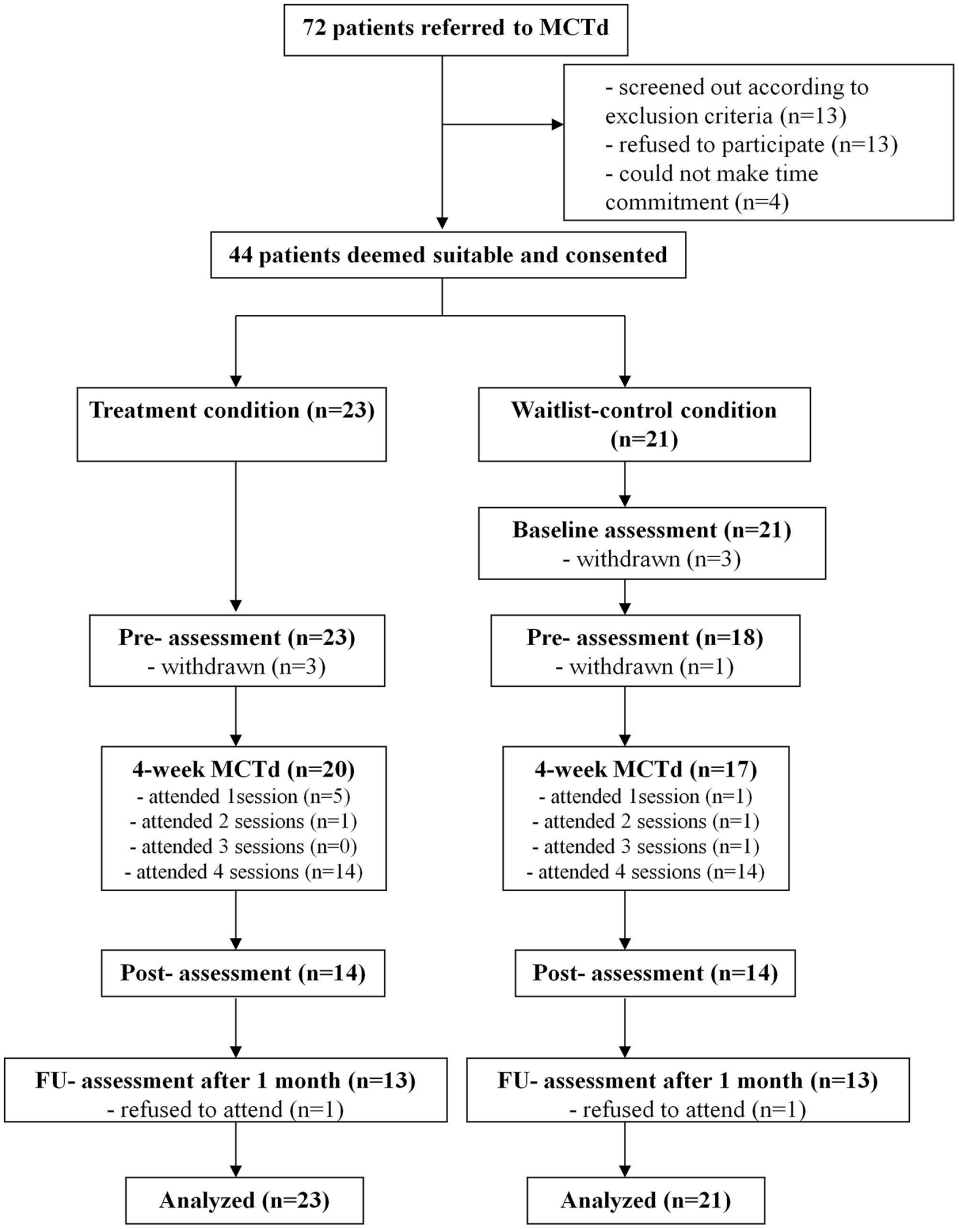
**Consort diagram of the study design**.

In this randomized wait-list controlled study, consented participants were randomized into the MCTd condition or a wait-list condition (see **Figure 1** for the CONSORT diagram). Assessment took place before treatment, at the completion of treatment, and 4 weeks after (i.e., follow-up). The wait-list control group had an additional baseline assessment at the beginning of the waiting period (i.e., 4 weeks before the pre-treatment assessment).

All assessments were administered by a research assistant blinded to group allocation.

#### Metacognitive Training for Delusions

The MCTd included modules 2 and 7 (JTC), module 3 (Changing beliefs), and module 5 (Memory – Overconfidence in errors) of the original MCT program (manual and session materials downloadable from http://www.uke.de/mct). The sessions (1-hour each) took place once a week, over four consecutive weeks. All the MCTd sessions were delivered by a qualified clinical psychologist who specializes in psychosis. The therapist received MCT training from the original authors of the MCT, and received regular training and supervision from the first author. Following the MCT manual, each MCTd session consists of (i) general introduction and normalization of the reasoning bias, (ii) illustration of the bias using interactive exercises where the participant was asked to make judgments and interpret events, and (iii) discussion on the link between the reasoning bias and delusional thinking, supported by scientific evidence.

Informed by studies using the individualized versions of MCT (e.g., Moritz et al., 2011b; Waller et al., 2011; Garety et al., 2014), we have made several adaptations to the MCTd. Firstly, MCTd was delivered in a one-on-one format, allowing the therapist to pace the sessions according to the individual’s learning.

Secondly, part 3 of the session (i.e., discussion of the link between the reasoning bias and delusional thinking) was extended and enhanced in MCTd to identify specific examples of the patient’s own experiences where the reasoning bias is in action. Whilst MCT groups also encourage participants to link their learning to their daily life, the discussion is more generic. In MCTd, the discussion bridged the reasoning bias with actual experiences and beliefs (which may include delusional beliefs if the patient was ready to discuss that), consolidating the individual’s reflection on how the bias affected the way s/he interprets his/her own idiosyncratic experiences, worries, symptoms, and daily life problems. This adaptation renders the format of MCTd more comparable to that of MCT+ than to MCT.

Thirdly, to deepen the individual’s learning after the session, each participant was given a handout which consists of (i) a summary of the learning points in the session, (ii) pictures abstracted from the session slides that would remind participants of the key points, and (iii) two reflective questions. Question 1 concerns the participant’s own recent experiences on which the reasoning bias had an impact. Question 2 concerns strategies that the participant could practice in a similar situation in the future. Participants were told how to fill out the handout before the end of each session, and were asked to bring back the completed handout for discussion in the next session.

While the Traditional Chinese and Simplified Chinese versions of MCT are available on the MCT website, no local adaptations have been made to the content of the modules. According to Bernal et al. (2009, p. 362), cultural adaptation for treatment protocols includes not just translation, but a “systematic modification… to consider language, culture, and context in such a way that is compatible with the client’s cultural patterns, meaning, and values.” Such adaptation is particularly important for MCT because the therapeutic process relies heavily on the discussion of daily life experiences commonly observed in the community. Without a clear understanding of the scenarios used, participants might encounter difficulty comprehending what the scenarios intend to explain.

**FIGURE 2:**
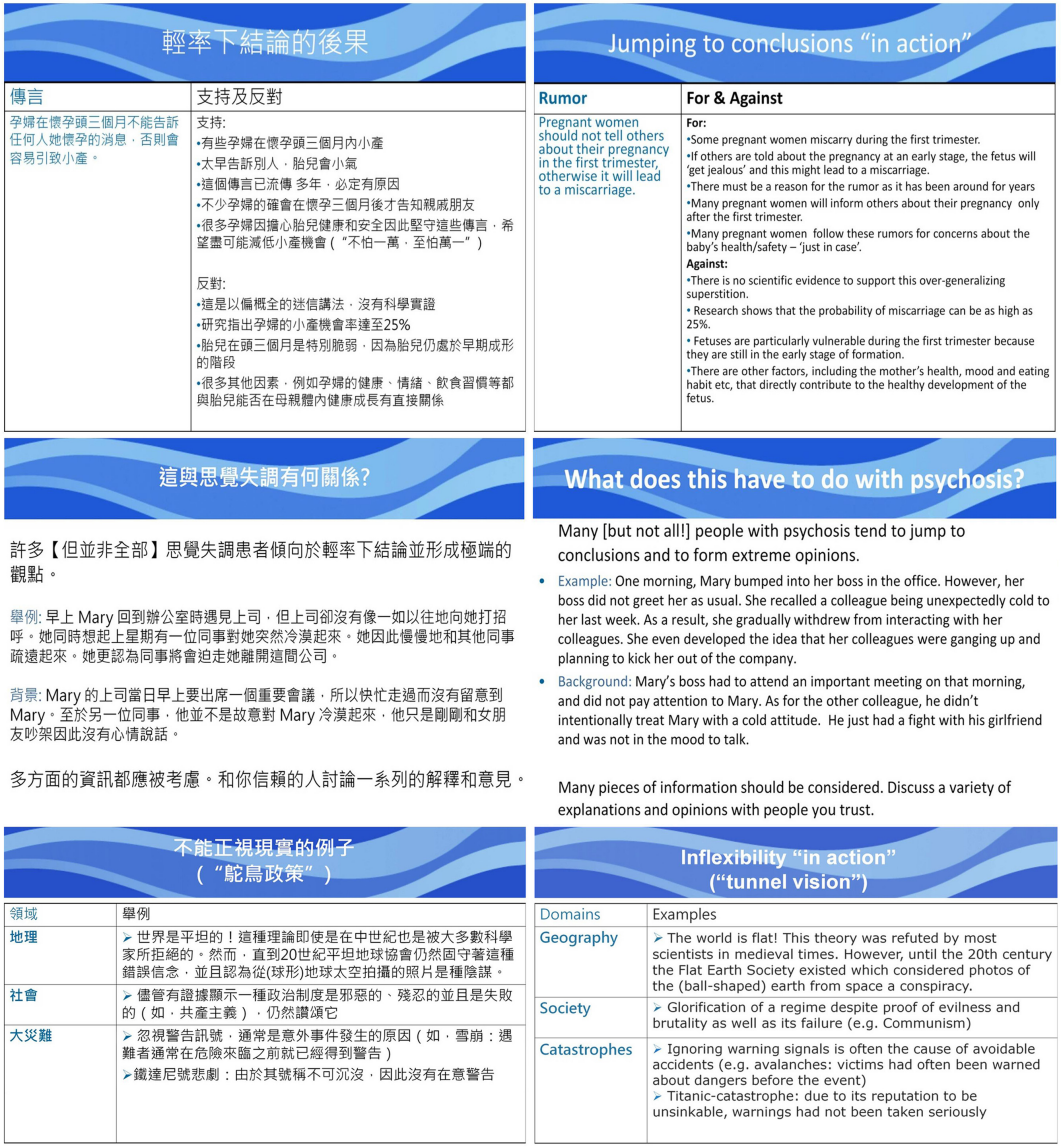
**Samples of modified slides used in Metacognitive Training for Delusions (MCTd)**.

The authors of the present study went through the presentation slides systematically to identify scenarios and examples that appeared to be more familiar to the West than to the Hong Kong Chinese population. We then came up with a list of alternative examples that were deemed culturally neutral or more relevant to the local Hong Kong Chinese service users. Two patients with delusions were invited to comment on the familiarity and relevance of the original (Western) scenarios and the newly suggested (local) scenarios. Based on their suggestions, some of the slides were revised. For example, the conspiracy theory about Paul McCartney’s death, which was used to illustrate JTC in the original MCT, was substituted by a classic local myth about keeping pregnancy secretive during the first trimester so as to avoid miscarriage. Another scenario in the original MCT using the story of a man who believed himself to be the successor of the Prussian throne to explain JTC was replaced with a scenario about a lady misperceiving her colleagues’ non-verbal cues as persecutory threats. In addition, following the pilot patients’ comments and suggestions, some wordings on the presentation slides and handouts were adapted (e.g., “Stalinism” was replaced with “Communism,” formal Chinese words were replaced with more colloquial spoken Cantonese words). To finalize the adaptation for the actual study, two clinical psychologists and one psychiatrist (all Cantonese speakers) were invited to review the overall presentation and clarity of the modified version of presentation slides and handouts. Some of the modified slides are shown in **Figure 2**.

#### Wait-List Condition

Participants on the wait-list condition received MCTd 4 weeks after baseline, provided by the same therapist. During the 4-week waiting period, participants would receive TAU, which includes outpatient assessment, psychiatric follow-up and pharmacological intervention (antipsychotics). There was no formal psychological treatment during the waiting period.

### Measures

#### Clinical Rating Scales (Baseline, Pre-Treatment, Post-Treatment, and Follow-Up)

The PANSS Kay et al. (1987) is a 30-item, seven-point (1–7) rating scale developed for assessing phenomena associated with schizophrenia. Symptoms over the past week are rated. The PANSS has four scores: positive (seven items), Negative (seven items), General psychopathology (16 items), and Total (30 items). Good psychometric properties for the PANSS have been reported (Kay et al., 1987, 1989; Kay, 1990).

The Psychotic Symptom Rating Scales (PSYRATS; Haddock et al., 1999) is a 17-item, five-point (0–4) scale measuring multiple dimensions of auditory hallucinations and delusions. Symptoms over the past week are rated. Two scores are obtained: auditory hallucinations (11 items) and Delusions (6 items). The PSYRATS has good psychometric properties (Haddock et al., 1999) and has been used as outcome measure for psychological interventions for psychosis (Lewis et al., 2002; Durham et al., 2003).

#### Reasoning Bias Measures (Baseline, Pre-Treatment, Post-Treatment, and Follow-Up)

The Maudsley Assessment of Delusions Scale (MADS; Wessely et al., 1993; Garety et al., 2005) is a standardized interview that assesses eight dimensions of delusional experience. The belief maintenance section of the MADS inquires about the evidence for the delusion. In this section, the participant is asked whether it is possible for him/her to be mistaken about the evidence for the delusion. The interviewer also presents a hypothetical but plausible piece of evidence in contradiction to the delusion. Whether the participant reports that this would reduce conviction in the delusion is recorded. Responses to these questions have been used to assess belief flexibility in large-scale studies (Freeman et al., 2004; Garety et al., 2005).

To assess the JTC bias, two versions of the beads task (Garety et al., 1991) were used. In the original version of the beads task, individuals are presented with two jars each containing 100 colored beads. One of the jars contains 85 beads of color A and 15 beads of color B, while the other jar contains 85 beads of color B and 15 beads of color A. Individuals are told that beads will be drawn, one at a time, from one of the jars, and will then be replaced. They can see as many beads as they like before deciding from which jar the beads are drawn. Apart from the original version (consisting of 85:15 beads of two colors; Garety et al., 1991), this study also included the more difficult version (consisting of 60:40 beads of two other colors; Dudley et al., 1997). The variable is the number of beads the participant requests to view before his/her decision. The “JTC” bias is defined as making a decision with two beads or fewer (Garety et al., 2005).

For both beads tasks, once participants have decided on the jar that the beads were drawn from, they were asked to rate on their confidence in their decision. This procedure had been used in McKay et al. (2006) and Warman (2008).

#### Estimate Level of General Intelligence (Baseline or Pre-Treatment Only)

All participants were administered a three-subtest short form of the Taiwanese version of Wechsler Adult Intelligence Scale (Third Edition; WAIS-III; Weschler et al., 2002), the version commonly used in Hong Kong. This short form (Vocabulary, Matrix Reasoning, and Information) had been reported to have high reliability and validity (Sattler, 2008). The sum of the age-scaled scores was used as an estimate of the participant’s general intellectual functioning.

#### Subjective Satisfaction and Effectiveness (Post-Treatment Only)

Upon completion of treatment, participants were asked to rate on eight questions about satisfaction and subjective efficacy of the treatment (e.g., “The training was useful, interesting and sensible”; “I would recommend the training to others,” “I found the training easy to grasp and enjoyable,” “I could apply what I have learnt in daily life,” “MCT helped reduce my emotional, behavioral and cognitive distress” and “MCT is an important part of my treatment plan”). The total satisfaction score ranged from 8 to 40.

### Statistical Analysis

For hypothesis 1, descriptive statistics of treatment compliance and subjective satisfaction ratings were reported, using data of patients on both randomized conditions, following the intention-to-treat principle.

For hypotheses 2 and 3, changes in primary outcome measures were analyzed in two stages. The first stage of analysis aimed to test the hypotheses that change during MCTd is greater than change during the waiting period (i.e., TAU). In this stage, differences in outcomes between pre- and post-treatment assessments in the treatment condition were compared against differences between baseline and pre-treatment assessments in the wait-list condition. For changes in continuous variables (PANSS and PSYRATS scores), the outcome variables were entered as DVs, Time was entered as within-subject IV and randomized condition was entered as between-subject IV in mixed-design ANOVAs. For categorical measures (data-gathering and belief flexibility), changes in outcome variables were coded into binary variables (i.e., 1 = improvement; 0 = no improvement) for binary logistic regression. In order to compare the effect of MCTd and TAU, effect sizes of the outcome variables [Cohen’s (1988) *d* and phi coefficients for continuous and categorical variables respectively] were calculated using change scores after MCTd for the treatment condition and change scores after TAU for the wait-list condition.

The second stage of analysis tested the MCTd treatment efficacy across time points, using data from both conditions. We tested changes across pre-treatment, post-treatment and follow-up assessments. In this stage of analysis, mixed-design ANOVAs were calculated for continuous outcome measures, with planned Bonferroni-corrected contrasts. Outcome variables were entered as DVs, Time was entered as within-subject IV and randomized condition was entered as between-subject IV. Cochran’s *Q*-tests were performed for categorical outcome measures, with *post hoc* McNemar tests. In this two-stage analysis, all available data were used. If a participant missed one assessment time point, that time point would be dropped whereas the remaining time points were retained in the analysis.

To examine the role of JTC and belief flexibility as mediators of treatment (Hypothesis 4), linear regression models were tested using data from participants who completed the pre-treatment and post-treatment assessments. In these respective models, treatment change in reasoning biases (JTC and belief flexibility) was IV and treatment change in delusions (severity and conviction) was DV, controlling for pre-treatment level of delusions.

Statistical analysis was conducted on the IBM SPSS Statistics for Windows, Version 21.0 (IBM Corp. Released, 2012).

## Results

### Demographic and Clinical Data

The sample consisted of 44 Chinese participants, including 24 (54.5%) male and 20 (45.5%) female. Their mean age was 33.91 years (SD = 11.94). Psychiatric diagnoses, according to the Diagnostic and Statistical Manual of Mental Disorders, Fourth Edition, Text Revision (DSM-IV-TR; American Psychiatric Association [APA], 2000), were available from 41 patients’ medical records as follows: schizophrenia 25 (61.0%); delusional disorder 8 (19.5%); schizoaffective disorder 1 (2.4%); psychotic disorder not otherwise specified 3 (7.3%); severe depression with psychotic symptoms 3 (7.3%); bipolar disorder 1 (2.4%). All but one participants (*N* = 43) were on antipsychotic treatment at the time of recruitment: 42 patients were on atypical antipsychotics (Risperidone, Olanzapine, Quetiapine, Amisulpiride, Clozapine, and Aripiprazole) and one was on Flupentixol. Mean dose of antipsychotics in chlorpromazine equivalents (CPZ; Woods, 2003; Andreasen et al., 2010) was 280.74 mg/day (SD = 216.43).

The overall mean PANSS scores were as follows: positive 20.68 (SD = 4.85), negative 14.70 (SD = 5.29), general 39.75 (SD = 9.20), total 75.14 (SD = 16.32). Mean score of the PANSS delusions item (P1) was 5.30 (SD = 0.88). A majority (*n* = 36; 81.82%) of patients scored 3 or above on the PANSS suspiciousness item (P6), whereas 15.91% (*n* = 7) scored 3 or above on the PANSS grandiosity item (P5). On PSYRATS, mean delusions score was 17.64 (SD = 2.72) and mean conviction score was 3.27 (SD = 0.69), with 40 patients (90.91%) reporting conviction of 50% or above and 17 patients (38.6%) reporting full conviction (100%).

Twenty-three participants were randomized to the treatment condition, and 21 to the wait-list control condition. As shown in **Table 1**, the randomized groups were matched on gender, age, level of education, and sum of WAIS-III subtest scaled scores (*p* > 0.05). The groups did not differ in primary psychiatric diagnosis (*p* > 0.05). The groups were also matched on number of admissions, mean dose of antipsychotics in chlorpromazine equivalents, and most PANSS scores, including suspiciousness and grandiosity items (*p* > 0.05). However, the treatment group had a higher PANSS total score than wait-list controls (*p* = 0.032).

**Table 1:** Demographic and clinical variables at baseline.

Measures	Treatment condition (*N* = 23)	Wait-list control condition (*N* = 21)	Group difference
Gender	Male 12 Female 11	Male 12 Female 9	χ^2^(1, *n* = 44) < 0.01, *p* = 0.951
Age	32.35 (12.87)	35.62 (10.89)	*U* = 190.50, *p* = 0.230
Years of education	(*n* = 21) 11.67 (2.78)	(*n* = 21) 12.81 (3.19)	*U* = 171.50, *p* = 0.209
Sum of WAIS subtest scaled scores	(*n* = 22) 21.55 (6.04)	(*n* = 18) 24.72 (9.76)	*t*(38) = -1.26, *p* = 0.215
PANSS total	80.13 (16.80)	69.67 (14.22)	*t*(42) = 2.22, *p* = 0.032
PANSS positive	21.48 (5.33)	19.81 (4.20)	*t*(42) = 1.15, *p* = 0.259
PANSS delusions	5.35 (0.89)	5.24 (0.89)	*t*(42) = 0.41, *p* = 0.684
Number of admissions	1.62 (0.35)	0.93 (0.20)	*t*(41) = 0.73, *p* = 0.472
Dosage of antipsychotics (CPZ)	217.36 (172.37)	336.79 (248.41)	*U*(42) = 177.00, *p* = 0.127

### Treatment Compliance and Satisfaction

Metacognitive training for delusions attendance rates are shown in **Figure 1**. Independent-samples *t*-tests revealed no significant group difference in attendance rate (*p* > 0.05). Number of sessions attended was not associated with age, years of education, sum of WAIS-III subtest scores, family income, or PANSS scores (*p* > 0.05).

As shown in **Table 2**, participants reported a high level of subjective satisfaction of the intervention. The randomized groups did not differ significantly on the overall level of subjective satisfaction (*p* > 0.05).

**Table 2:** Ratings of subjective satisfaction towards Metacognitive Training for Delusions (MCTd).

Item (score range: 1–5)	Treatment condition (*N* = 12)		Wait-list condition (*N* = 14)		Whole sample (*N* = 26)	
	Mean	SD	Mean	SD	Mean	SD
(1) This intervention is useful.	4.25	0.62	4.21	0.43	4.23	0.51
(2) I can apply what I have learnt in daily life.	4.08	0.52	4.21	0.58	4.15	0.54
(3) This intervention is an important part of my treatment plan.	4.00	0.00	4.07	0.48	4.04	0.34
(4) This intervention helps to reduce my emotional, cognitive and behavioral distress.	4.17	0.39	4.29	0.47	4.23	0.43
(5) This intervention is interesting.	4.08	0.29	3.93	0.62	4.00	0.49
(6) This intervention is easy to understand.	4.00	0.43	3.93	0.62	3.96	0.53
(7) I enjoyed the intervention.	4.25	0.45	4.21	0.43	4.23	0.43
(8) I would recommend this intervention to others.	3.92	0.67	4.21	0.70	4.08	0.69

### Efficacy on Severity and Conviction of Delusions

Levels of severity and conviction of delusions and their test statistics are shown in **Table 3**.

**Table 3:** Comparisons of severity and conviction of delusions across time points.

Measures	Wait-list condition				Treatment condition			Overall change between pre-treatment, post-treatment, and follow-up assessments	*Post hoc* pairwise comparisons
	Baseline	Pre	Post	Follow-up	Pre	Post	Follow-up	Effect of time	
PANSS positive	19.81 (4.20)	18.65 (5.27)	12.36 (4.34)	11.54 (5.13)	21.48 (5.33)	13.64 (4.57)	13.23 (6.50)	Wilks’ λ = 0.31, *F*(2,23) = 26.09, *p* < 0.001, observed power = 1.00, $\eta_{p}^{2}$ = 0.69	Post–Pre (*p* < 0.001) FU-Pre (*p* < 0.001) FU-Post (*p* = 0.917)
PANSS delusions	5.24 (0.89)	5.41 (1.18)	2.93 (1.27)	2.54 (1.56)	5.35 (0.89)	3.36 (1.45)	3.15 (1.91)	Wilks’ λ = 0.22, *F*(2,23) = 40.10, *p* < 0.001, observed power = 1.00, $\eta_{p}^{2}$ = 0.78	Post–Pre (*p* < 0.001) FU-Pre (*p* < 0.001) FU-Post (*p* = 0.262)
PSYRATS delusions	17.62 (2.50)	16.35 (5.18)	7.29 (4.97)	6.77 (5.48)	17.61 (2.92)	8.93 (5.76)	8.00 (7.04)	Wilks’ λ = 0.20, *F*(2,23) = 45.65, *p* < 0.001, observed power = 1.00, $\eta_{p}^{2}$ = 0.80	Post–Pre (*p* < 0.001) FU-Pre (*p* < 0.001) FU-Post (*p* = 1.000)
PSYRATS conviction	3.33 (0.58)	3.29 (0.59)	1.93 (1.44)	2.23 (1.36)	3.22 (0.80)	2.21 (1.12)	1.85 (1.57)	Wilks’ λ = 0.46, *F*(2,23) = 13.26, *p* < 0.001, observed power = 0.99, $\eta_{p}^{2}$ = 0.54	Post–Pre (*p* < 0.001) FU-Pre (*p* = 0.001) FU-Post (*p* = 1.000)

Mixed-design ANOVAs comparing change before and after MCTd in the treatment condition and change before and after TAU in the wait-list condition revealed significant Group × Time interaction effects on PANSS positive score (*p* < 0.001), PANSS delusions score (*p* < 0.001), PSYRATS delusions score (*p* < 0.001), and PSYRATS conviction score (*p* = 0.008), indicating that changes during MCTd were significantly different from changes during TAU in these outcome measures. Group × Time interaction effects on these PANSS and PSYRATS scores remained significant after controlling for baseline PANSS total score (*p* < 0.05). Compared to change after TAU, there was a large effect size of change after MCTd for PANSS positive score (*d* = -1.71), PANSS delusions score (*d* = -1.86), PSYRATS delusions score (*d* = -1.63), and PSYRATS delusional conviction (*d* = -0.98).

Mixed-design ANOVAs for the entire sample revealed that there was a significant change over time (from pre-treatment to follow-up) in PANSS positive score, PANSS delusions score, PSYRATS delusions score, and PSYRATS conviction score (*p* < 0.001; see **Table 3**). There was no significant Group × Time interaction for these PANSS and PSYRATS scores (*p* > 0.05), indicating that changes after treatment did not differ between the two randomized conditions.

*Post hoc* Bonferroni tests revealed significant improvements between pre- and post-treatment assessments, and between pre-treatment and follow-up assessments, in PANSS positive score, PANSS delusions score, PSYRATS delusions score, and PSYRATS conviction score (*p* < 0.05, see **Table 3**).

### Effect on Data Gathering and Belief Flexibility

#### Change in Data Gathering

Change in JTC bias is shown in **Table 4**.

**Table 4:** Comparisons of jumping to conclusions bias and over-confidence in decisions across time points.

Measures	Wait-list condition				Treatment condition			Overall change between pre-treatment, post-treatment, and follow-up assessments	*Post–hoc* pairwise comparisons
	Baseline	Pre	Post	Follow-up	Pre	Post	Follow-up	Effect of time	
85:15 beads task – DTD	2.52 (4.16)	2.53 (3.74)	3.79 (5.45)	5.08 (7.15)	1.52 (0.73)	2.29 (1.86)	3.00 (2.16)	Wilks’ λ = 0.74, *F*(2,23) = 3.95, *p* = 0.034, observed power = 0.65, $\eta_{p}^{2}$ = 0.26	Post–Pre (*p* = 0.080) FU-Pre (*p* = 0.062) FU-Post (*p* = 0.799)
60:40 beads task – DTD	3.14 (4.48)	3.71 (5.06)	5.21 (6.60)	6.69 (7.13)	2.00 (1.24)	3.57 (3.03)	4.08 (3.12)	Wilks’ λ = 0.67, *F*(2,23) = 5.57, *p* = 0.011, observed power = 0.81, $\eta_{p}^{2}$ = 0.33	Post–Pre (*p* = 0.021) FU-Pre (*p* = 0.013) FU-Post (*p* = 0.913)
85:15 beads task – JTC (%)	80.95	88.24	71.43	69.23	87.06	64.29	46.15	Cochran’s *Q* (2,26) = 9.46, *p* = 0.009	Post–Pre (*p =* 0.070) FU-Pre (*p* = 0.008) FU-Post (*p* = 0.687)
60:40 beads task – JTC (%)	66.67	58.82	50.00	38.46	73.91	50.00	38.46	Cochran’s *Q* (2,26) = 7.43, *p* = 0.024	Post–Pre (*p* = 0.146) FU-Pre (*p* = 0.057) FU-Post (*p* = 0.500)
85:15 beads task – confidence	75.43 (25.44)	75.63 (23.59)	65.29 (21.49)	78.77 (21.54)	66.67 (22.38)	63.93 (20.21)	74.23 (18.24)	Wilks’ λ = 0.76, *F*(2,21) = 3.38, *p* = 0.053, observed power = 0.57, $\eta_{p}^{2}$ = 0.24	Post–Pre (*p* = 0.753) FU-Pre (*p* = 1.000) FU-Post (*p* = 0.050)
60:40 beads task – confidence	65.00 (20.37)	63.75 (24.19)	57.86 (20.45)	60.77 (16.18)	56.67 (19.58)	60.00 (16.64)	66.54 (25.45)	Wilks’ λ = 0.97, *F*(2,21) = 0.36, *p* = 0.705, observed power = 0.10, $\eta_{p}^{2}$ = 0.03	Post–Pre (*p* = 1.000) FU-Pre (*p* = 1.000) FU-Post (*p* = 1.000)

At baseline, the two randomized groups were not significantly different in the number of beads drawn to decision (DTD) or confidence ratings in their decisions on either beads task (*p* > 0.05). Percentage of participants showing a JTC bias (defined by DTD ≤ 2) was also not different between groups on either beads task (*p* > 0.05).

Mixed-design ANOVAs and binary logistic regression comparing change before and after MCTd in the treatment condition and change before and after TAU in the wait-list condition revealed no significant group difference in changes in DTD, JTC bias or decision confidence on either beads task (*p* > 0.05). Therefore, changes in these data-gathering measures were not significantly different following MCTd or TAU.

Mixed-design ANOVAs for the entire sample revealed that there was a significant change over time in DTD on both beads tasks (*p* < 0.05; see **Table 4**). Change in decision confidence was at a trend level for the 85:15 task (*p* = 0.053) and was not significant for the 60:40 task (*p* > 0.05). Cochran’s *Q*-tests showed significant reductions over time in JTC bias on both beads tasks (*p* < 0.05).

*Post hoc* Bonferroni tests revealed significant improvements between pre- and post-treatment assessments, and between pre-treatment and follow-up assessments, in DTD on the 60:40 beads task (*p* < 0.05, see **Table 4**). Decision confidence did not change significantly during the treatment, but increased (at a marginally significant level) on the 85:15 task during the follow-up period. *Post hoc* McNemar tests revealed that difference between pre-treatment and follow-up assessments of the JTC bias was significant on the 85:15 task (*p* = 0.008) and marginally significant on the 60:40 task (*p* = 0.057).

#### Change in Belief Flexibility

**FIGURE 3:**
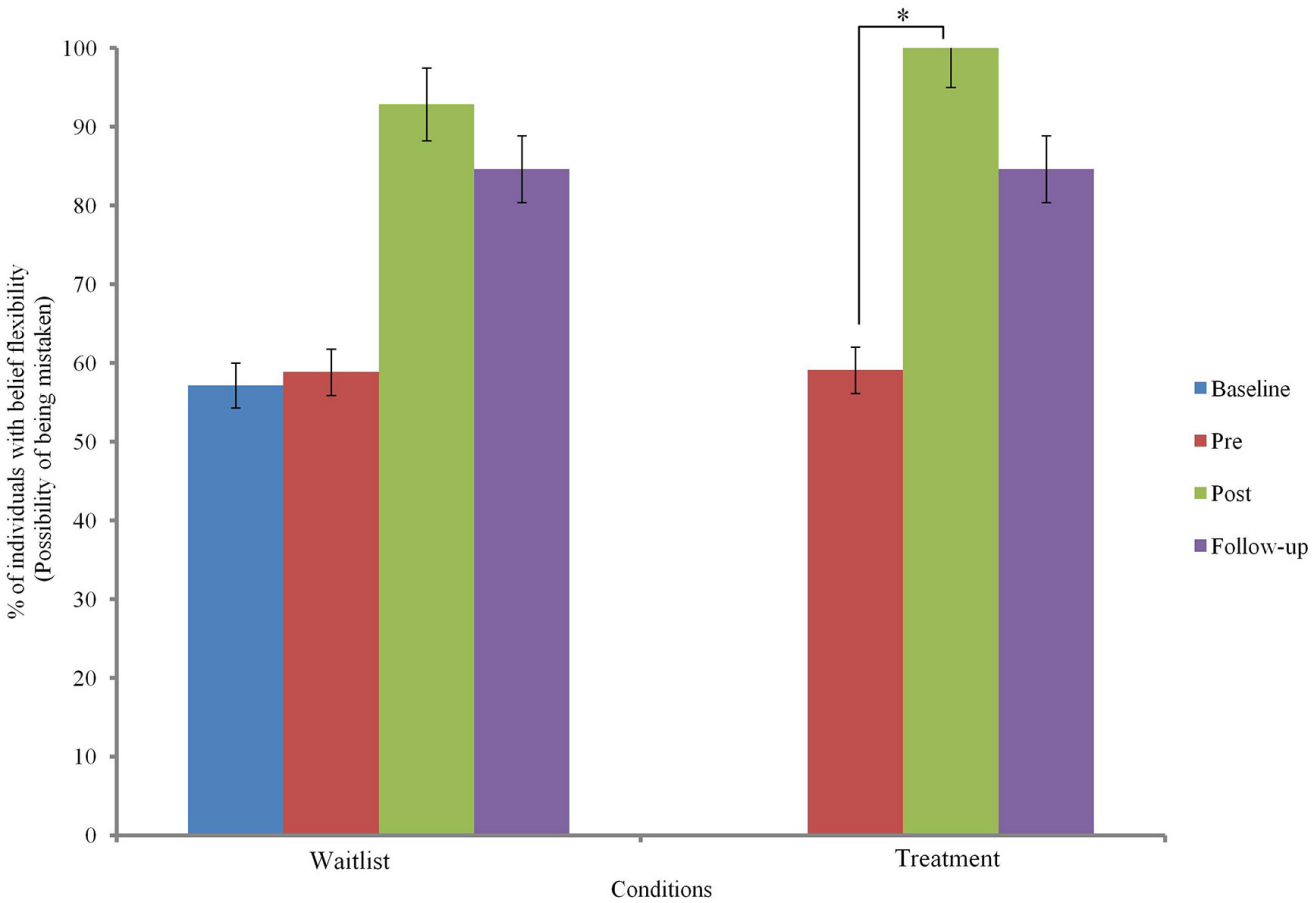
**Possibility of being mistaken (PM) across groups.** ^∗^ indicates *p* < 0.05.

**FIGURE 4:**
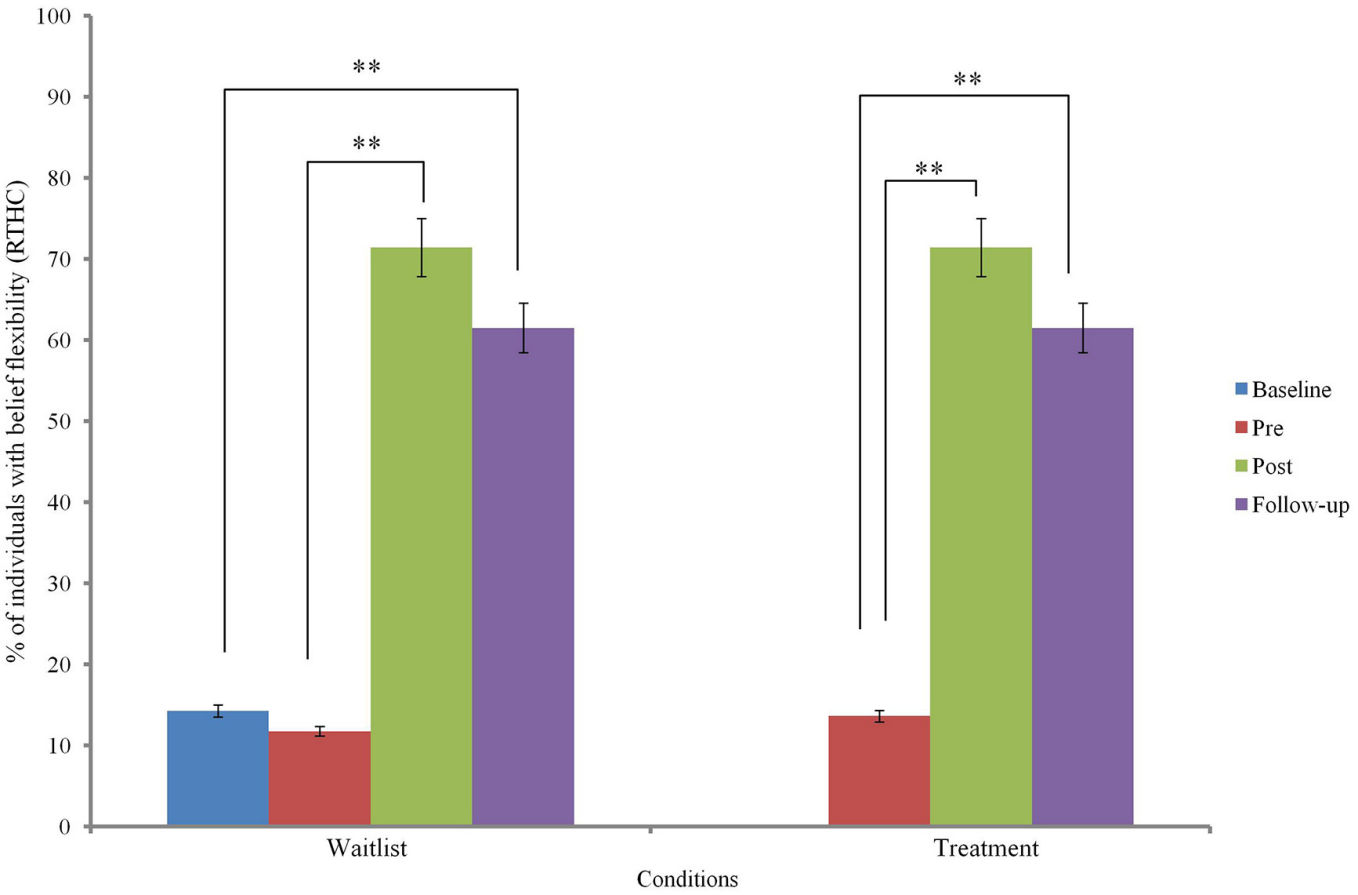
**Reaction to hypothetical contradiction (RTHC) across groups.** ^∗∗^ indicates *p* < 0.01.

Changes in belief flexibility are shown in **Table 5** and **Figures 3** and **4**.

**Table 5:** Comparisons of belief flexibility across time points.

Measures	Wait-list condition				Treatment condition			Overall change between pre-treatment, post-treatment, and follow-up assessments	*Post hoc* pairwise comparisons
	Baseline	Pre	Post	Follow-up	Pre	Post	Follow-up	Effect of time	
PM (% showing flexibility)	57.1	58.8	92.9	84.6	59.1	100	84.6	Cochran’s *Q* (2,26) = 14.00, *p* = 0.001	Post–Pre (*p* = 0.004) FU-Pre (*p* = 0.031) FU-Post (*p* = 0.250)
RTHC (% showing flexibility)	14.3	11.8	71.4	61.5	13.6	71.4	61.5	Cochran’s *Q* (2,26) = 26.00, *p* < 0.001	Post–Pre (*p* < 0.001) FU-Pre (*p* = 0.001) FU-Post (*p* = 0.375)

At baseline, there was no significant group difference in the possibility of being mistaken (PM) measure or the reaction to hypothetical contradiction (RTHC) measure of belief flexibility (*p* > 0.05).

Binary logistic regression comparing change before and after MCTd in the treatment condition and change before and after TAU in the wait-list condition indicated a significant group difference in RTHC change [β = 2.93, SE = 0.96, Wald χ^2^ (1) = 9.37, *p* = 0.002] but not in PM change [β = 1.73, SE = 0.93, Wald χ^2^ (1) = 3.48, *p* = 0.062]. This indicates that change in RTHC during MCTd was significantly different from change in RTHC during TAU. The group difference in RTHC change remained significant after controlling for baseline PANSS total score (*p* < 0.05). Compared to change after TAU, there was a large effect size of change after MCTd for both PM (φ = 0.92) and RTHC (φ = 1.46).

Using data of the entire sample, Cochran’s *Q*-test revealed a significant improvement in both PM and RTHC across three time points (*p* < 0.05). *Post hoc* McNemar tests showed significant improvements in both belief flexibility measures between pre- and post-treatment assessments and between pre-treatment and follow-up assessments (*p* < 0.05; see **Table 5**).

### Cognitive Processes as Mediators of Treatment Effect on Delusions

Regression analyses revealed that treatment change in DTD or confidence ratings on either beads task did not predict change in PANSS and PSYRATS scores (*p* > 0.05). Improvement in JTC bias (yes/no) on either beads task did not predict improvement in PANSS or PSYRATS scores (*p* > 0.05).

Treatment change in PM (yes/no) did not significantly predict change in PANSS and PSYRATS scores (*p* > 0.05). However, treatment change in RTHC (yes/no) predicted changes in PANSS positive (β = -0.50, SE = 1.78, *t* = -2.95, *p* = 0.007), PANSS delusions (β = -0.40, SE = 0.48, *t* = -2.23, *p* = 0.035), PSYRATS delusions (β = -0.56, SE = 1.67, *t* = -3.46, *p* = 0.002), and PSYRATS conviction (β = -0.40, SE = 0.48, *t* = -2.23, *p* = 0.035). After controlling for baseline scores, treatment change in RTHC remained a significant predictor of treatment changes in PANSS positive (β = -0.41, SE = 1.41, *t =* -3.05, *p* = 0.005), PSYRATS delusions (β = -0.59, SE = 1.72, *t* = -3.54, *p* = 0.002), and PSYRATS conviction (β = -0.37, SE = 0.48, *t* = -2.08, *p =* 0.048). Participants who improved in RTHC had more reduction in positive symptoms and delusions after treatment.

## Discussion

This study evaluated the effect of a four-session MCTd on reducing delusions and improving data-gathering and belief flexibility. We found (i) a large and significant effect of MCTd in improving positive symptoms and delusions, (ii) a large and significant effect in improving one of the measures of belief flexibility, and (iii) evidence for improvement in belief flexibility as the mediator for symptom improvement.

Psychosis is complex with patients experiencing highly varied symptom profile and treatment needs. Adopting a single-symptom approach, MCTd focused specifically on reasoning biases that have been shown to be closely associated with the pathogenesis of delusions. Despite its brevity, MCTd showed promise for symptom-specific improvement. We found statistically and clinically significant treatment effects in reducing positive symptoms and delusions, which were maintained after 1 month post-treatment. Our large treatment effect sizes for overall symptomatology, delusional severity and conviction were larger or comparable with previous MCT based studies (Moritz et al., 2011b; Erawati et al., 2014; Garety et al., 2014; Waller et al., 2015). MCTd was half the duration of the original MCT, and 40% the duration of the individualized MCT+ for psychosis. MCTd sessions rely less substantially on discussing and challenging the idiosyncratic content of delusions than in MCT+ or CBTp. The large effect sizes of delusion change achieved by MCTd suggests that MCT, with its modular structure each focusing on a specific reasoning bias, can be provided in a more cost-effective manner by matching selected treatment modules with the individual’s symptoms and treatment needs. This treatment approach can be strengthened by identification of the symptom structure of psychosis (e.g., Potuzak et al., 2012; Russo et al., 2014) and research that links specific psychotic symptoms to specific reasoning biases (e.g., So et al., 2010). The level of subjective satisfaction and attendance rate (84.5%) reported by our sample, which consists of patients with a high level of delusional conviction, also showed promise for patients’ acceptance of this form of intervention.

As a process-based intervention, we found an improvement in reasoning biases that MCTd was meant to ameliorate, especially belief flexibility. Following MCTd, patients became more flexible in accommodating new information that contradicts their delusional beliefs. Treatment effect in increasing perceived possibility of being mistaken did not reach statistical significance by a small margin. However, the percentage of participants who considered the possibility that their belief might be wrong increased from <60% before treatment to >90% after treatment, and the large effect size supported clinical significance of the change. While an improvement in PM was also reported in the MRTP trial (Garety et al., 2014), improvement in RTHC was reported for the first time in this study. More importantly, improvement in RTHC significantly predicted improvement in positive symptoms and delusions. This indicates that the ability to accommodate disconfirmatory evidence may be a mediator of treatment-induced delusion change. This is consistent with previous finding that patients who have better belief flexibility are more responsive to cognitive therapy for delusions (Chadwick and Lowe, 1994; Garety et al., 1997; Kuipers et al., 1998). Altogether, these findings suggest that MCTd is effective in ameliorating delusions, potentially via increasing belief flexibility. MCTd can also be used to prepare patients who may not yet be ready for CBTp (Waller et al., 2015).

In our study, change in belief flexibility was evident right after MCTd, whereas change in belief flexibility took place after 2 weeks of post-MRTP homework exercises (Garety et al., 2014). This raises the possibility that the combination of structured training and individualized homework exercises is beneficial to drawing links between the training and patients’ daily life applications, hence augmenting treatment effect.

Treatment effect on data-gathering was more modest than on belief flexibility, and took place more slowly. We found no significant difference in JTC change between MCTd and TAU. However, when participants on both conditions were pooled for analysis, there was a significant post-treatment increase in number of draws to decision and a decrease in prevalence of JTC bias. Therefore, effect of MCTd on JTC may be subject to sample sizes, and hence replications of results are warranted. We found that change in JTC did not mediate symptom improvement following MCTd. This is consistent with Menon et al. (2008), which reported that change in JTC did not mediate delusion change following antipsychotics, and with previous studies that showed a closer association between delusions (especially delusional conviction) with BF than with JTC (So et al., 2010, 2012). Despite a small change in data-gathering, our results add to the accumulating evidence that individualized variants of MCT (including MRTP and MCT+) show promise for JTC improvement, which is not achieved by antipsychotics or CBT (Peters and Garety, 2006; So et al., 2012). Future research on these interventions with a larger sample and a longer follow-up may unveil a treatment effect that potentially takes place over a longer period of time.

This study had a number of limitations. Firstly, the 4-week follow-up period was relatively short for evaluating longer-term improvement in more trait-like variables such as JTC. Secondly, the small sample size limited the power of the mediation analysis and did not allow for more sophisticated approaches such as Baron and Kenny’s (1986) causal-steps approach or Sobel first-order test (Fritz and MacKinnon, 2007). Thirdly, our sample had a range of psychiatric diagnoses and symptom profiles, introducing the issue of heterogeneity. However, the two randomized groups were matched on psychiatric diagnosis, as well as on major clinical and demographic variables. Where the groups were not matched, i.e., on the PANSS total score, the baseline score was controlled for in the main analyses. Fourthly, psychiatric diagnosis was obtained from patients’ medical notes. It would be preferred if a structured diagnostic interview, including a more comprehensive assessment of delusional subtypes were included. Lastly, we did not include measures of neurocognitive abilities that may affect patients’ performance on the reasoning measures, such as working memory. Likewise, we may have missed other processes that are also important in maintaining delusions, such as emotional processes and coping behavior. Against these caveats, this study provided support for MCTd, a locally adapted brief reasoning training, in improving delusions and associated reasoning biases. With its theoretical basis, structured format, user-friendly manuals, and free availability of numerous translations, MCT and its variants invite larger scale outcome evaluations for wider dissemination across populations.

## Conflict of Interest Statement

The authors declare that the research was conducted in the absence of any commercial or financial relationships that could be construed as a potential conflict of interest.
